# Excipient-Free Inhalable Microparticles of Azithromycin Produced by Electrospray: A Novel Approach to Direct Pulmonary Delivery of Antibiotics

**DOI:** 10.3390/pharmaceutics13121988

**Published:** 2021-11-23

**Authors:** Beatriz Arauzo, Tania B. Lopez-Mendez, Maria Pilar Lobera, Javier Calzada-Funes, Jose Luis Pedraz, Jesus Santamaria

**Affiliations:** 1 Instituto de Nanociencia y Materiales de Aragón (INMA), CSIC-Universidad de Zaragoza, 50009 Zaragoza, Spain; beaagm@unizar.es (B.A.); plobera@unizar.es (M.P.L.); jcalzadafunes@unizar.es (J.C.-F.); 2 Department of Chemical and Environmental Engineering, Campus Río Ebro-Edificio I+D, University of Zaragoza, 50018 Zaragoza, Spain; 3 Networking Research Center on Bioengineering, Biomaterials and Nanomedicine, CIBER-BBN, 28029 Madrid, Spain; tblopez01@gmail.com; 4 NanoBioCel Research Group, School of Pharmacy, University of the Basque Country (UPV/EHU), 7, 01006 Vitoria-Gasteiz, Spain; 5 Bioaraba, NanoBioCel Research Group, 01009 Vitoria-Gasteiz, Spain

**Keywords:** azithromycin, electrospray, pulmonary administration, dry powder, microparticles

## Abstract

Inhalation therapy offers several advantages in respiratory disease treatment. Azithromycin is a macrolide antibiotic with poor solubility and bioavailability but with a high potential to be used to fight lung infections. The main objective of this study was to generate a new inhalable dry powder azithromycin formulation. To this end, an electrospray was used, yielding a particle size around 2.5 µm, which is considered suitable to achieve total deposition in the respiratory system. The physicochemical properties and morphology of the obtained microparticles were analysed with a battery of characterization techniques. In vitro deposition assays were evaluated after aerosolization of the powder at constant flow rate (100 L/min) and the consideration of the simulation of two different realistic breathing profiles (healthy and chronic obstructive pulmonary disease (COPD) patients) into a next generation impactor (NGI). The formulation was effective in vitro against two types of bacteria, *Staphylococcus aureus* and *Pseudomonas aeruginosa*. Finally, the particles were biocompatible, as evidenced by tests on the alveolar cell line (A549) and bronchial cell line (Calu-3).

## 1. Introduction

Lung delivery of aerosolized drugs to treat respiratory diseases is possibly attracting more attention than ever in a context of global pulmonary infections. This is not surprising, since local administration to the lungs for the treatment of respiratory tract diseases has many advantages compared to other administration routes. The internal lung high surface area (>100 m^2^) allows drugs to be distributed and absorbed efficiently, with high effective drug concentrations reaching the lung. This allows for the decrease of the total administered dose, systemic exposure and toxicity, and, as a consequence, the main adverse effects generated by drugs, in particular antibiotics [1,2]. These features are especially interesting in the treatment of pulmonary infections, since local delivery of antibiotics in the lungs could allow for early bacterial eradication and, thus, enable a shorter treatment of minimal systemic exposure. Furthermore, aerosolized antibiotics are able to reduce airway bacterial density due to the high concentrations of drug in the action site, helping to avoid the appearance of resistances [3]. However, formulation design, inhalation device and particle size are key issues in determining the aerosol performance of the drug [4]. In particular, achieving a dry powder formulation of the right particle size is highly challenging, as severe problems such as particle size dispersion and agglomeration must be avoided. Several techniques have focused on the generation of antibiotic formulations with the aim of avoiding these problems; among them, we can highlight supercritical antisolvent precipitation (amoxicillin or cefuroxime) [5,6], spray-drying (ciprofloxacin) [7] or freeze-drying (clarithromycin) [8].

Azithromycin (AZT) is one of the safest antibiotics for respiratory diseases according to the World Health Organization (WHO) [9]. Several studies have shown azithromycin’s clear potential as a drug for the treatment of respiratory infections. AZT has the ability to accumulate and penetrate in lung tissues, thus higher concentrations can be achieved [10]. It seems that passive and active mechanisms are involved. The first mechanism is due to the transmembrane transport and capture by protonation in acid organelles. The second mechanism is promoted by transporter protein (s), which is dependent on factors such as cell viability or temperature [11]. This accumulation results in a long half-life (68–79 h) of the drug. Among its characteristics, the excellent penetration in lung tissues and the sustained drug concentration by tissues and cells, makes the azithromycin a good candidate for the treatment of respiratory infections [12].

AZT belongs to the macrolide group with a broad-spectrum action. Its mechanism involves inhibiting protein synthesis by reversible binding to the 50S ribosomal subunits [13]. It presents activity against both Gram–negative and Gram–positive pathogens [14], such as *Staphylococcus aureus* and *Pseudomonas aeruginosa*. These bacteria are responsible for several chronic lung infections, especially in patients with impaired immune system or who are suffering from damaged mucosa such as cystic fibrosis (CF) disease [12,15]. As a consequence of these lung infections, a further deterioration of lung function appears. Moreover, this drug exhibits anti-inflammatory, muco-regulatory and anti-biofilm activities. Due to its anti-inflammatory activity, AZT has been authorised for treatment of community acquired pneumonia and exacerbations of chronic obstructive pulmonary disease (COPD) by the FDA [16]. Presently, AZT is administered orally or systemically, although it shows poor bioavailability and a lack of diffusion through the tissues. Data on efficacy and safety on longer treatment has revealed important side effects, such as gastrointestinal symptoms or hearing impairment, that are associated with the use of this antibiotic by oral route [17]. In this way, an alternative delivery of AZT microparticles by inhalation would enable the reduction of side effects associated with the current administration routes.

While there are different devices for pulmonary administration, perhaps the most notable are dry powder inhalers (DPIs) due to their easy handling, portability and drug state, since the powder form is more stable than the liquid pharmaceutical presentation [18]. The optimal particle size to obtain a complete pulmonary deposition is within the range of 0.5–5 µm. Many DPI formulations employ a combination of fine drug particles and carrier excipient (lactose and mannitol are typically used), with the goal of improving the flow and ensuring the consistency of aerosol performance. However, the overall efficiency of administration is still low. It has been found that only 10–15% of the drug is able to reach the deep lung, 20% of the drug inhaled is swallowed and the rest of the drug (around 65%) is not even released from the carrier due to interparticulate adhesive forces [19]. Moreover, there are multiple clinical challenges that have to be faced by DPIs, particularly related to variable patient factors such as age, clinical condition and inspiratory flow [20]. Here, choosing the correct carrier excipient is critical, since it should help particle aerosolization, and after inhalation, it should be easily dispersed in the lung and then eliminated. Otherwise, due to inhalation of a large amount of drug/excipient powders, local adverse effects may be caused, such as bronchospasm and coughing. In the case of antibiotic administration, the presence of excipients as carriers or in charge of other functions represents a significant drawback since the antibiotic dose becomes limited by the presence of these additional materials [21,22]. For these reasons, we aimed to develop an excipient-free dry powder formulation seeking to circumvent the above-mentioned problems of limited antibiotic dose and local conditions generated by the slow solubility of carriers, as well as achieving high bioavailability and reproducibility of the production process.

Electrospray (ES) is an advanced technology to produce particles with a well-defined size. In this case, a drug or polymeric solution is subjected to a large potential difference that breaks up the liquid in drops that, upon solvent evaporation, yield the drug as dry powder particles [23]. Microparticle engineering by electrospray has many advantages for DPI formulations. A variety of parameters (e.g., solvent, concentration, potential, flow rates) can be tuned to obtain the desired size range, and in many cases, it is possible to avoid the use of surfactant, stabilizers and excipients [24]. Here an organic drug solution was electrosprayed for the generation of carrier excipient– free AZT formulation for DPI devices.

The electrospraying process yields amorphous particles that present significant advantages regarding solubility-related bioavailability issues, facilitating solubility and thus, improving the bioavailability of the drug, as shown in previous studies [25]. The antibiotic microparticles produced, not only exhibited high antimicrobial activity against two different strains of bacteria, but also optimal response in two different breathing profiles, healthy and COPD patients, when introduced into a DPI device. In this study, anatomical structures (such as the Alberta throat) and breathing profiles have been used to solve the in vitro-in vitro correlation problems.

## 2. Materials and Methods

### 2.1. Materials

Azithromycin dihydrate (AZT) was supplied by Alfa Aesar. Phosphate buffered saline tablets (PBS) pH 7.4, ethanol, acetone, chloroform and methanol were purchased from Sigma–Aldrich (Burlington, MA, USA). Glycerol and Brij-35 were provided by Sharlau (Barcelona, Spain) and Fagron (Rotterdam, The Netherlands) respectively. Acetonitrile (ACN) was purchased from WVR (Avantor, Arnhem, The Netherlands). UPLC grade water was obtained from a Milli-Q Advantage A10 System with resistivity of 18.2 mΩ (Merk Millipore, Darmstadt, Germany).

Tryptone soy broth (TSB) and tryptone soy agar (TSA) were supplied by Conda-Pronadisa (Torrejón de Ardoz Madrid, Spain). Eagle’s Minimum Essential Medium (EMEM) was purchased from ATCC. Dulbecco’s modified Eagle’s medium (DMEM w/stable Glutamine), Dulbecco’s Phosphate Buffered Saline (DPBS), penicillin, streptomycin and amphotericin B were bought in Biowest. Fetal bovine serum (FBS) was supplied by Thermo Fisher Scientific (Waltham, MA, USA).

Bacteria strains, *Staphylococcus aureus* (ATCC 25923; Ielab, Las Atalayas, Alicante, Spain) and *Pseudomonas aeruginosa* (ATCC 10145; Ielab, Spain). Cell line A549 (ATCC–CCL–185) was a gift from P. Martin-Duque and used between passages 25–32 and Calu-3 (ATCC-HTB-55) was acquired in ATCC and used between passages 24–30.

### 2.2. Electrospray

A Yflow 2.2D-500 electrospinner (Coaxial Electrospinning Machines/R&D Microencapsulation, Málaga, Spain) was employed to produce microparticles. Several solutions of different concentration of AZT (10 to 100 mg/mL) and four solvents (ethanol/acetone (1/1), ethanol, acetone and chloroform) were tested. These solutions were conducted at flow rates from 0.5 mL/h until 2.5 mL/h controlled by a syringe pump. The positive electrode was connected to the needle (0.6 mm of inner diameter) and a stationary collector plate was joined to negative electrode. All experiments were conducted in a closed chamber at room temperature, relative humidity of 25–50% and under atmospheric pressure. The tip-to-collector distance (H) was fixed at 10 cm in all experiments and voltage values were between 15 to −8 kV.

### 2.3. Scanning Electron Microscopy (SEM)

Morphology and size distribution of electrosprayed microparticles were analysed by scanning electron microscopy (SEM). Observations were conducted on a FEI Inspect field emission instrument at accelerating voltages between 5–10 k. Samples were sputter-coated with palladium (Pd) (Leica EM ACE200 coater) prior to examination.

### 2.4. X-ray Diffraction (XRD)

Crystallinity of raw material and microparticles were determined by X-ray diffraction (Empyrean, Malvern Panalytical, Malvern, United Kingdom). A Cu-Kα radiation source and a D/tex ultra-detector were used. The samples were spread on a glass slide and placed in the measuring chamber. XRD scanning was set as 5 to 40° 2Ɵ at 0.013/200 s with a voltage of 45 kV and a current of 40 mA.

### 2.5. Differential Scanning Calorimetric (DSC)

Thermal analysis and phase transition measurements were performed on DSC822^e^ (Mettler Toledo, Columbus, OH, USA) equipped with T-Zero technology and an automated computer-controlled RSC-90 cooling accessory. Approximately 5 mg samples were packed into aluminium crucible lids pans (100 µL size) which were then hermetically sealed with the Tzero hermetic sealer (Mettler Toledo, Columbus, OH, USA). Nitrogen gas was used as purging gas for the DSC analysis. The reference pan was an empty hermetically sealed aluminium one. The samples were heated from 30 °C to 400 °C at a heating scan rate of 10 °C/min with a gas purge flow rate of 50 mL/min.

### 2.6. Solid State Fourier Transform Infrared Spectroscopy (FTIR)

The raw material and electrospray microparticles were analysed using a Vertex-70 FTIR spectrophotometer (Bruker, Billerica, MA, USA) equipped with an attenuated total reflectance (ATR) sample stage. Each spectrum was analysed with a resolution 4 cm^−1^ in the range on 600–4000 cm^−1^ and 40-scan.

### 2.7. Drug Quantification

The limit of detection (LOD) is defined as the lowest concentration of the analyte that can be detected, under the stated experimental conditions. On the other hand, the limit of quantification (LOQ) is defined as the lowest concentration in a sample that may be measured with an acceptable level of accuracy and precision under the stated experimental conditions [26,27].

In order to calculate these parameters within the conditions of the performed analysis, both the standard deviation of y-intercepts (σ) and the slope (*s*) from the calibration curve were employed as follows in (Equations (1) and (2)):

(1) $$\mathrm{LOD}=3\times \left(\frac{\sigma }{s}\right)$$

(2) $$\mathrm{LOQ}=10\times \left(\frac{\sigma }{s}\right)$$

According to this, LOQ was estimated as 1.001 ppm and LOD as 0.300 ppm.

Azithromycin analysis was performed on a Waters ACQUITY system H-Class, which consisted of a binary pump, an autosampler, a column thermostat and a photodiode array (PDA) detector. This system is coupled to a single quadrupole mass spectrometer with an electrospray ionization (ESI) ACQUITY QDa mass detector. Data acquisition and processing were performed using MASSLYNX software 4.1 (Waters Corporation, Milford, MA, USA), date of accession: 14 May 2021. Chromatographic separation was carried out using a CORTECS^®^ UPLC C18 column (90 Å, 1.6 µm 2.1 × 100 mm, from WATERS) at 40 °C, under an isocratic flow of 0.5 mL/min containing 70% acetonitrile and 30% milli Q water. For both solvents, 0.1% *v*/*v* of 10 mM ammonium chloride in ammonium solution was employed as a mobile phase modifier.

PDA detector was employed to monitor absorbance from azithromycin at a wavelength of 210 nm during analysis time. However, ACQUITY QDa mass detector was used to quantify azithromycin according to the most abundant ion, corresponding to a *m/z* ratio of 375.26 ([M-2H^+^]^2+^). Under these conditions, the retention time of azithromycin was 5.75 min.

A calibration curve of azithromycin was obtained using commercial standards of azithromycin (r^2^ > 0.998) within the required concentration range (0.5–10 µg/mL). Both samples and standards were filtered with 0.22 µm Nylon filters before injecting in UPLC system. Quantitative analysis was performed in triplicate.

### 2.8. Equilibrium Solubility 

The equilibrium azithromycin solubility was determined in PBS medium adjusted to different pH, PBS (7.4), slightly acidic PBS (6.5) and basic PBS (8.2). An excess of azithromycin (3 ± 0.250 mg/mL of each formulation) was added to 1 mL of dissolution media and continuous stirring at 37 ± 1 °C (Thermo-Shaker, Biosan, Riga, Latvia) for 24 h and 48 h. Buffer media (PBS) was used instead of water to simulate the lung lining fluid to study the solubility of microparticles at different pH. After, suspensions were centrifuged at 12,000 rpm for 10 min and the supernatant was filtered using a 0.22 µm nylon syringe filter, azithromycin concentrations were quantified by UPLC.

### 2.9. Aerosol Properties 

#### 2.9.1. Aerodynamic Particle Size 

***Pharmacopeia assay.*** The powder dispersion performance of particles was analysed using a next generation impactor (NGI) equipped with mouthpiece, stainless-steel induction port, pre-separator and steel NGI insert impactor stages (NGI, Copley Scientific Limited, Nottingham, UK). The NGI was also completed with a Copley HCP5 vacuum pump as well as Copley TPK2000 critical flow controller. The airflow rate was calibrated with a Copley DMF2000 flow meter before each experiment. 15 mg of AZT MPs powder were loaded into jelly hard capsules and were subsequently released into the NGI using a dry powder inhalation device (Breezhaler^®^) with a flow rate of 100 L/min determined by the resistance of the device [28] (Table S1 in Supplementary Information (SI)) and a conducting time of 2.4 s. The effective aerodynamic cut off diameter for each impactor stage at 100 L/min was set following the manufacturer´s instructions: Stage 1 (6.12 µm), Stage 2 (3.42 µm), Stage 3 (2.18 µm), Stage 4 (1.31 µm), Stage 5 (0.72 µm), Stage 6 (0.40 µm) and Stage 7 (0.24 µm).

Each stage and pre-separator were covered with a mix of Brij-35 (15% *w*/*w*), glycerol (34% *w*/*w*) and ethanol absolute (51% *w*/*w*). In each measurement, a total number of 3 different capsules (15 mg/capsule) were used in order to obtain an enough amount of dry powder deposited on the different parts of the NGI and each experiment was repeated 3 times for statistical analysis of the results (3 capsules × 3 measurements, 9 capsules). After each experiment, the dry powder deposited on the different NGI components, capsules and inhalation device was recovered separately in ethanol and afterwards analysed by UPLC.

The emitted fraction (EF) (Equation (3)), fine particle fraction (FPF) (Equation (4)) and respirable fraction (RF) (Equation (5)) were calculated by the following equations:

(3) $$\mathrm{EF}\;\left(\%\right)=\left(\frac{\mathrm{ED}}{\mathrm{TD}}\right)\times \;100$$

(4) $$\mathrm{FPF}\;\left(\%\right)=\left(\frac{\mathrm{FPD}}{\mathrm{ED}}\right)\times \;100$$

(5) $$\mathrm{RF}\;\left(\%\right)=\left(\frac{\mathrm{FPD}}{\mathrm{DD}}\right)\times \;100$$

The term emitted dose (ED) refers to the difference between the initial powder mass and the powder mass detected after the aerosolization process (except capsules and device). Total dose (TD) is the amount of powder loaded into the capsules. Fine particle dose (FPD) refers to the dose deposited on impactor stages 2–7. Fine particle fraction (FPF) was determined as the drug mass deposited in the NGI (aerodynamic diameter ≤ 4.46 µm) over the emitted dose. And finally, deposited dose (DD) represents the dose deposited on impactor stages 1–7.

***Simulated breath profiles tests***. With the objective of obtaining a more precise estimation of the AZT MPs deposition in vitro in respiratory system, the breathing simulator model BRS 2100 was used. The constant flow at 100 L/min (used as standard in the United States pharmacopeia assay [28]) was replaced by a respiratory profile from either healthy or COPD patients. Both respiratory profiles were obtained from bibliography [29,30] and they were introduced in the programme controlling the test flow (table parameters were described in Table S2 and Figure S1 [30,31]). For these experiments, a variable flow was used and the NGI was connected to the breathing simulator, and to Alberta throat (replacing the induction port), mixed inlet and pre-separator were also used as in the assays carried out previously (experimental device was described in Figure S2). As described above, each experiment was repeated 3 times (3 capsules × 3 measurements, 9 capsules) and each capsule was loaded with 15 mg of AZT MPs. As in the previous case, Breezhaler^®^ was used as inhaler device.

#### 2.9.2. Physical Particle Size

The volumetric particle size distribution was obtained with a laser diffraction aerosol spectrometer (Spraytec, Malvern Instruments, UK) coupled to the inhalation cell and Copley HCP5 vacuum pump [28,32]. For each measurement a capsule loaded with 15 mg of powder was used, and the aerosol was generated by passing the airflow (100 L/min) through the sample. Each experiment was repeated 3 times for statistical analysis of the results (1 capsule × 3 measurements, 3 capsules). When the aerosol cloud was generated, the particles from the device went through the inhalation cell. The aerosol was detected by the optical system allowing the determination of the particle size distribution (measurement time: 2.4 s). The main values obtained were, the volumetric median diameter (VMD = Dv10; Dv50; Dv90) and span value (Equation (6)).

(6) $$\mathrm{Span}\;=\frac{\mathrm{Dv}90-\mathrm{Dv}10}{\mathrm{Dv}50}$$

### 2.10. Antibacterial Activity

Antimicrobial activity was evaluated in gram (+) bacteria model (*S. aureus*) and gram (−) bacteria model (*P. aeruginosa*). AZT MPs were employed to determine the minimum inhibitory concentration (MIC) and minimum bactericidal concentration (MBC) values. 

Both strains were grown for 16 h in TSB in a shaker Innova^®^ 40 (New Brunswick Scientific, Enfield, CT, USA) 150 rpm and 37 °C. Finally, 10^8^–10^9^ colony-forming units/mL (CFU/mL) were obtained. Experiments were made with a final concentration of ~10^5^ CFU/mL.

AZT MPs as dry powder were suspended in liquid growth medium (TSB) and sonicated for 5 min before being diluted to perform the different bactericidal tests. Subsequently, in the case of *S. aureus*, different concentrations of AZT MPs (from 0.50 to 64.00 µg/mL) were inoculated. In the case of *P. aeruginosa* concentrations from 16.00 to 512.00 µg/mL were employed, according to previous assays showing that each of the bacterial strains required a different concentration of azithromycin to reach the MIC and MBC.

#### 2.10.1. Optical Density (OD_600_) Measurements

Optical densities of pathogenic bacteria were measured at 600 nm (Implen^TM^ OD600 DiluPhotometer^TM^; ThermoFisher Scientific, Waltham, MA, USA) (adapted from Liu et al. [33]) with the objective to monitor their growth. *S. aureus* and *P. aeruginosa* contained an initial concentration of ~10^5^ CFU/mL while they were put in contact with AZT MPs. These mixed suspensions were kept in a shaker at 37 °C and 150 rpm. During essays, optical density was measured at different time points, up to 24 h.

#### 2.10.2. Agar Dilution Method

The number of CFU/mL was quantified in order to evaluate the antimicrobial activity of MPs [34]. Bacterium culture was diluted up to ~10^5^ CFU/mL and then inoculated with different concentrations of microparticles. Bacteria were kept in a shaker at 37 °C and 150 rpm during 24 h. Then, bacteria suspensions were diluted in PBS and seeded in Petri plates with TSA at 37 °C for 24 h. 

The MBC was determined by testing the concentration that showed no visible bacteria growth (the lowest concentration that kills > 99.95 of the bacteria).

### 2.11. In Vitro Cytotoxicity Studies

#### 2.11.1. Cell Culture Conditions

Cell line A549 from human alveolar epithelium was cultured in Dulbecco´s Modified Eagle´s Medium (DMEM; Gibco, Invitrogen, Waltham, MA, USA) and Eagle´s Minimum Essential Medium (EMEM; ATCC, Barcelona, Spain) was used to culture human bronchial epithelium cell line Calu-3. The medium in both cultures was supplemented with 10% *v*/*v* fetal bovine serum (FBS) and a mixture of antimitotic-antibiotic, penicillin (60 µg/mL), streptomycin (100 µg/mL) and amphotericin B (0.25 µg/mL).

The cytotoxicity potential of the raw AZT and MPs was evaluated with the Alamar Blue (AB) assay. 1 × 10^4^ cells/well in 100 µL were seeded onto 96-well plates and allowed to attach for 24 h in a CO_2_ incubator. Raw AZT and AZT MPs were suspended in culture medium, resulting in AZT concentrations of 0.1 to 1000 μg/mL. Cells incubated in DMEM or EMEM without particles were used as controls.

#### 2.11.2. Alamar Blue Assay

The metabolic activity of cells was measured 24 h and 48 h after treatment with raw AZT and MPs with the colorimetric AB assay. The medium with particles was removed and the cells were washed twice with DPBS and then incubated with fresh culture medium supplemented with the AB reagent (10% *v*/*v*; incubation at least 1 h at 37 °C and 5% CO_2_). The fluorescence displayed was recorded at λ_530_ nm excitation and λ_590_ nm emission in a microplate reader (Multimode Synergy HT Microplate Reader; Biotek, Winooski, VT, USA). The viability was calculated by linear interpolation of the mean fluorescence values (MFV) from the cells treated with raw AZT and MPs versus the untreated one (Equation (7)):

(7) $$\%\;\mathrm{Cell}\;\mathrm{viability}\;=\frac{\mathrm{MFV}\;\mathrm{of}\;\mathrm{treated}\;\mathrm{cells}}{\mathrm{MFV}\;\mathrm{of}\;\mathrm{control}\;\mathrm{cells}}\times 100$$

To determine the toxicity threshold, the ISO 10993-5 norm was used (biological evaluation of medical devices–Part 5: Tests for in vitro cytotoxicity), which considers a material as non-cytotoxic when cellular viability is >70%.

## 3. Results and Discussion

### 3.1. Production of Dry Microparticles

When particles are produced by electrospray, different parameters have an effect on morphology and size distribution of the resulting nano or microparticles. We have mainly focused on the concentration, solvent and flow rate of the drug solutions, as they play a central role during particle formation. The conditions used in the different experiments performed (different solvents, concentrations and flow rates) as well as the particle sizes obtained, are summarized in Tables S3 and S4.

Selection of a suitable solvent is key, not only concerning its properties (viscosity, surface tension, dielectric constant, electrical conductivity) that have a direct influence on the electrospraying process, but also because of its influence on the shape of microparticles. Thus, a solvent with a fast evaporation may produce holes in the surface of microparticles and favour particle fragmentation. Moreover, it has been reported that, solvents with low conductivity values have more difficulties in increasing the coulomb electrostatic force and, consequently, in overcoming the surface tension, resulting in large particles being obtained [35]. Thus, to produce monodisperse particles, solvents with low conductivity values are preferred since high values can lead to elongated particles as a consequence of coulomb fission [36]. This can be observed in Figure S3, where particles obtained using ethanol and acetone showed holes in their surfaces as well as a higher size due to the high electrical conductivity value with respect to other solvents used. The best conditions were found with chloroform as solvent, since all the azithromycin microparticles (AZT MPs) displayed a round shape and an average size around 2 µm.

With chloroform as a solvent, the effect of the concentration of azithromycin on the final properties of microparticles was studied next. Table S4 shows the experimental parameters of the study (flow rate, concentration) and the particle sizes obtained. The flow range tested varied from 1 to 2.5 mL/h and different azithromycin concentrations were electrosprayed (1% to 10% *w*/*w*). A concentration of azithromycin as 10% *w*/*w* was selected, as it produced particles with a narrow size range and better morphology. The yield obtained was also very high, at around 85%.

**Figure 1:**
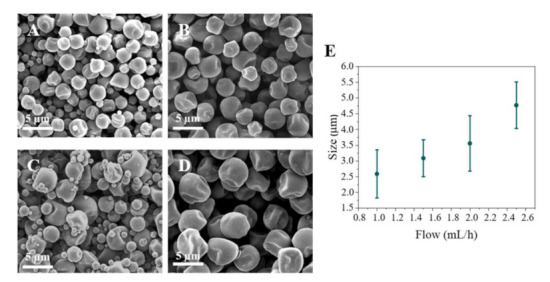
Particle size in function of flow (mL/h) at 10% *w*/*w* concentration with chloroform as solvent: (**A**) 1 mL/h; (**B**) 1.5 mL/h; (**C**) 2 mL/h; (**D**) 2.5 mL/h; (**E**) Influence of flow on particle size distribution (mean ± SD, *n* = 3).

The flow rate and concentration were important parameters regarding the particle size. Increasing the flow of drug solution generates a higher drop size, leading to larger particles. This can be observed in Figure 1, where an increase in the flow rate results in the generation of large size particles. The lowest flow rate studied (1 mL/h) allowed to generate size particles from 1 to 2 µm depending on the AZT concentration used. The optimal flow value to obtain the best homogeneity in particle size was set at 1.5 mL/h, giving the lowest size dispersion (Figure 1E).

**Figure 2:**
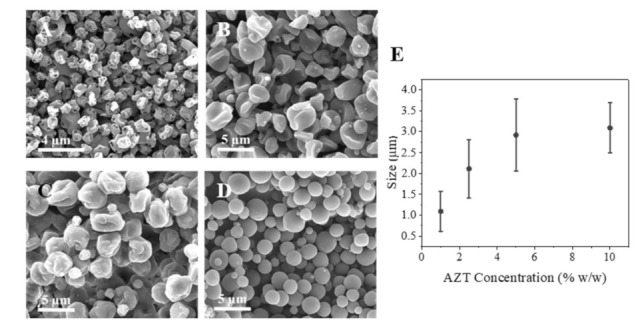
Particle size according to the concentration (% *w*/*w*) and the flow used (1.5 mL/h) with chloroform as solvent: (**A**) 1% *w*/*w*; (**B**) 2.5% *w*/*w*; (**C**) 5% *w*/*w*; (**D**) 10% *w*/*w*; (**E**) Influence of azithromycin concentration on particle size distribution (mean ± SD, *n* = 3).

The effect of the concentration was somewhat similar: a higher concentration leads to more material in the droplet, and thus to larger particles after solvent evaporation. Thus, in the case of the highest flow value tested (2 mL/h), the particle size increased from 2.50 to 3.50 µm for 1% *w*/*w* and 10% *w*/*w* AZT concentrations, respectively. Furthermore, for a given particle size the amount of solvent to be evaporated decreases as the AZT concentration increases, leading to better formed round shapes (Figure 2D).

### 3.2. Characterization of Microparticles 

FTIR was employed to analyse potential changes in azithromycin following electrospraying. To this end, the IR spectra of AZT MPs was compared with raw azithromycin (Figure S4). In the spectrum of raw azithromycin dehydrate, peaks at 3492 and 3559 cm^−1^ (grey area) were observed. These peaks can be assigned to the O–H stretching modes attributed to the presence of water in the crystal lattice. These peaks disappeared in the case of AZT MPs, which indicated the conversion of azithromycin to an anhydrous form.

**Figure 3:**
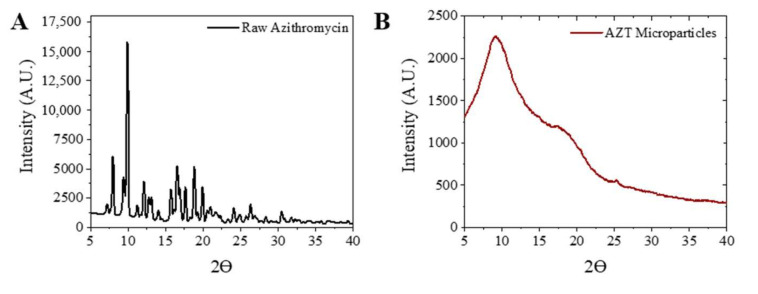
X-ray diffraction patterns: (**A**) raw azithromycin; (**B**) azithromycin microparticles.

To determine whether microparticle formulation prepared by electrospray has a different crystallinity structure than raw azithromycin, and in particular, whether a crystalline or amorphous form of the solid is collected after electrospraying, X-ray diffraction and DSC methods were used. Sharp diffraction peaks appear across the raw azithromycin XRD diffractograms indicating the crystalline structure of raw AZT (Figure 3A). In contrast, the AZT MPs collected after electrospraying only showed weak reflections (2Ɵ = 10° and 18°), which are related to the amorphous form (Figure 3B).

The thermograms obtained from raw AZT and AZT MPs are shown in Figure S5. Raw azithromycin presented two different endothermic peaks, one at 130–140 °C indicative of dehydration and recrystallization, and another peak at 250 °C due to the melting of the solid drug. In contrast, AZT microparticles only presented one peak at 250 °C, the peak at 130–140 °C disappeared, indicating that AZT became dehydrated during the electrospray process.

One of the main problems with azithromycin is its poor solubility in biological fluids, which causes a poor bioavailability, after its administration [37]. The amorphous forms tend to have advantages in this respect, especially for inhaled drug delivery, as it is often found that they increase the bioavailability for poorly soluble drugs by facilitating its solubility in biological fluids. The surface area of the amorphous form is generally higher than that of the crystalline form and it may enhance the dissolution rate. In addition, the crystalline form has a slower dissolution rate due to its more organized structure. As a consequence of this slower dissolution, particles are extensively removed from lungs by mucociliary clearance and the therapeutic effect may be reduced [38]. As a result, the amorphous form is preferred for use in DPIs in case of drugs that are poorly water soluble (like many antibiotics) [39].

**Figure 4:**
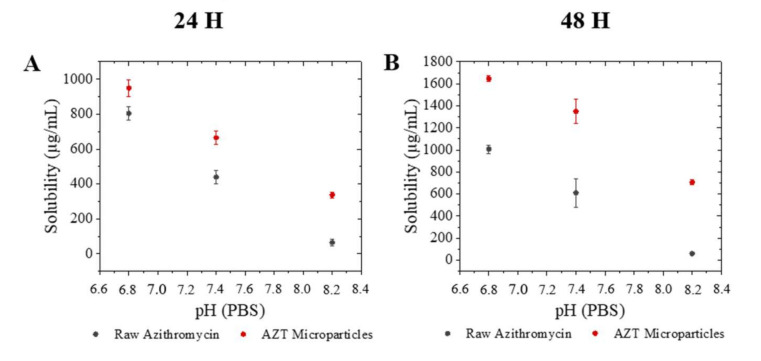
Equilibrium solubility: Raw azithromycin and azithromycin microparticles at different pH (6.8–8.2), (**A**) 24 h; (**B**) 48 h (mean ± SD, *n* = 3).

Indeed, the amorphous AZT microparticles showed a higher dissolution rate compared with its crystalline form. Solubility experiments were developed in dissolution with PBS (neutral, pH 7.4), acidic (HCl 0.1 M, pH 6.8) and basic (NaOH 1 M, pH 8.2). Figure 4 shows that in both materials the solubility decreases with the increase of pH but the solubility of AZT MPs was always higher than raw AZT. In addition to the intrinsically faster dissolution characteristic of amorphous forms, the higher solubility of the AZT microparticles is also aided by the morphology, as solid particles.

The higher solubility of AZT in acidic medium is beneficial to the capacity of macrolides to accumulate within phagocytes (among other cells). The phagolysosomal environment is characterized by low pH values (4.8–5.0) that are beneficial for drugs, such as azithromycin, in contrast with other antibiotics like fluoroquinolones (levofloxacin), which reduced their activity in acidic media [40].

### 3.3. In Vitro Aerosol Performance 

Newly developed DPI formulations must pass suitable tests to determine the most likely locations in the human respiratory tract where its particles will be deposited after inhalation. The breath simulation model developed by Copley^®^ is used to predict deposition of particulate drugs in different regions of the airway paths with more precision than the constant flow test required by the pharmacopeia test. Depending on the inspiratory flow rate, the patient is able to generate the amount of drug inhaled that is deposited in the lungs and can be modified. For this reason, two different breath profiles have been used, representative of a healthy and of a COPD patient, to compare the powder deposition under these two different situations.

The profiles used (Figure S1) are simulated breathing profiles based on the data of design studio composed by female and male patients, when using very low resistance DPIs (0.0202 KPa^0.5^/L^−1^ min). In the case of COPD profile, the patients had a clinical diagnosis of mild to severe COPD. The equations used to develop the profile are provided in Table S2. The data collected to obtain the values of PIFR (peak inspiratory flow rate), TPIFR (time at which PIFIR occurs) and V (inhaled volume) were evaluated for their dependence on airflow resistance (R).

One of the main differences between breathing profiles is the duration of the inspiration. In the case of a healthy patient profile, a slightly longer duration (2.5 s vs. 2.2 s) and a higher flow rate (Figure S1), 100 L/min against 72 L/min, is expected compared to a COPD patient.

**Figure 5:**
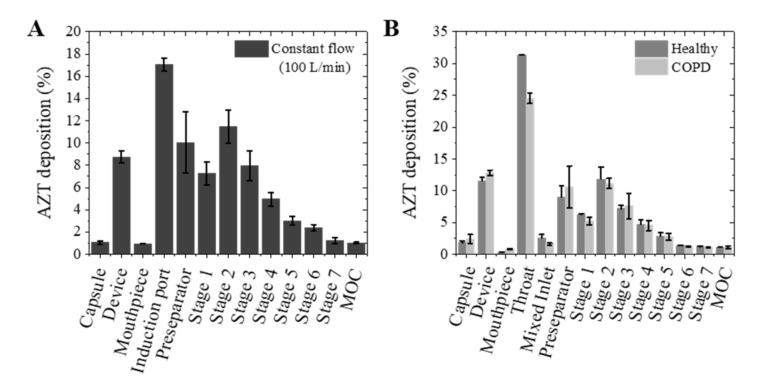
Aerodynamic particle size distribution: (**A**) mass deposited in the next generation impactor at constant flow (100 L/min); (**B**) mass deposited in the next generation impactor with respiratory profile from healthy and COPD patient (mean ± SD, *n* = 3).

In all the assays, AZT MPs formulation showed similar ED, around 85%, and FPF, around 15% (Table 1 and Figure S6). However, when breathing profiles were used, the drug distribution was clearly different. Thus, it could be observed in Figure 5 those higher quantities of AZT MPs are deposited in the capsule, device, mouth and throat when a breathing profile is used, in contrast with experiments carried out under a constant 100 L/min flow. Most of the dry powder stayed at stage 2 (3.42 µm), which corresponds well with the sample particle size (3.10 ± 0.60 µm).

According to the results of Figure 5, the COPD breathing profile had no effect on the total dose (TD) of MPs particles deposited. This means that the dose inhaled and the drug distribution in a COPD patient would be similar to that of a healthy patient. Breezhaler^®^ is a low resistance inhaler suitable for use by patients with a range of disease severities. So, one of the reasons for this could be that this device allows to generate adequate inspiratory flows to inhale the enough dose in a wide range of COPD severity patients [30]. In any case, a breathing profile allows the use of a realistic flow rate that changes with time, with peak flow rates of 101 L/min and 72 L/min for healthy and COPD group, respectively (Figure S1). These changes in flow rate affect the obtained results and are expected to provide a more realistic approach compared to the pharmacopeia test, with a constant flow rate of 100 L/min. As expected, the FPF values obtained in both breathing profiles are lower than that of the pharmacopeia test.

To understand the particle size distribution pattern that would be produced as a result of the airflow during the inhalation, particle size measurement of the aerosol clouds of the AZT MPs formulation was conducted using an in-situ particle size analyser (Spraytec^®^), coupled to the Breezhaler^®^ device. The microparticle powder was dispersed three times at a flow rate of 100 L/min. Figure S7 shown a bi-modal distribution of microparticles in the aerosol plume. The mode at the lower sizes was within the range of 1–15 µm, in the range of individual particles and small agglomerates. These sizes are expected to have nearly total deposition in the respiratory system [41]. In contrast, the second mode presented a wide distribution of particle agglomerates that the inhalation process (even at 100 L/min) is not able to separate individually, presenting a broad size distribution (30–500 µm). Table 2 shows the particle distribution as the volumetric median diameter and span values.

### 3.4. Antimicrobial Activity

To evaluate the antimicrobial activity of microparticles, the minimal inhibitory concentration (MIC) and minimal bactericidal concentration (MBC) were determined against *S. aureus* and *P. aeruginosa*, using optical density measurement (OD600 nm) and the result obtained was confirmed with the agar dilution method.

According to the literature, azithromycin presents MIC and MBC concentrations of 1–2 µg/mL and >64 µg/mL, respectively, for *S. aureus* [42]. For *P. aeruginosa,* the corresponding values are 128–256 µg/mL (MIC) and >512 µg/mL (MBC) [43].

**Figure 6:**
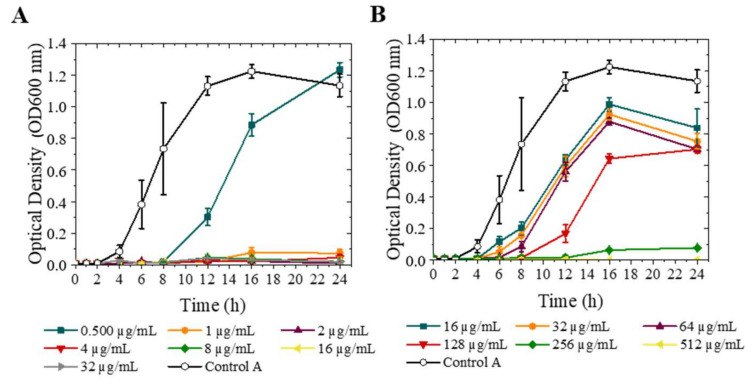
Antibacterial activity of azithromycin microparticles against *S. aureus* and *P. aeruginosa*. Control was C.A (bacteria untreated). OD_600_ (**A**) *S. aureus*; (**B**) *P. aeruginosa* (mean ± SD, *n* = 3).

Figure 6A,B show the growth curves of *S. aureus* and *P. aeruginosa* after the treatment with different concentrations of AZT MPs respectively. In both bacteria strains, it can be seen that the growth is inhibited when the AZT concentration approaches the MIC. The main reason could be the AZT post antibiotic effect (PAE) against some respiratory pathogens. PAE suppresses bacterial growth in many cases when the antibiotic concentration is below MIC value [42].

The same concentrations tested in OD600 nm assay were also studied by the agar dilution method and this confirmed the MIC and MBC values obtained for azithromycin microparticles by optical density measurements (see the CFU/mL obtained for each concentration in Figure S8A,B).

**Figure 7:**
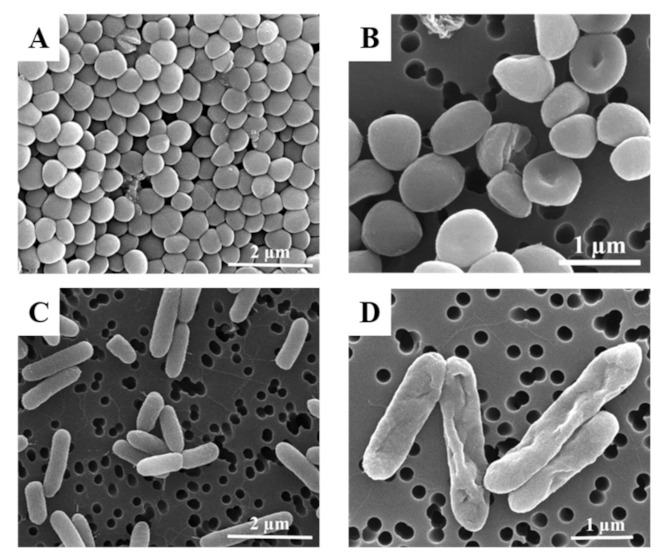
SEM images *S. aureus* (**A**) control; (**B**) after 24 h of treatment with AZT MPs; *P. aeruginosa* (**C**) control; (**D**) after 24 h treatment with AZT MPs at MIC concentrations.

Finally, different samples of both bacteria strains were observed by SEM after being treated with AZT microparticles (Figure 7B,D). *S. aureus* and *P. aeruginosa* controls showed a high concentration of viable bacteria displaying a normal morphology (Figure 7A,C). In contrast, in Figure 7B, *S. aureus* bacteria show a clear alteration in their morphology, presenting a rough outer membrane and in some cases, holes compared to the control sample. Ding et al. [44] showed in their study that Staphylococcus (gram (+) strain) could suffer from changes in the expression of ribosomal protein, producing an interruption or interference of protein synthesis and effects in biofilm formation. At the same time, the union to ribosomal units could be causing deformations in the wall of the bacteria due to the lack of protein. In the case of *P. aeruginosa* (gram (−) strain), the azithromycin directly affects the outer membrane (Figure 7D) where pits, dents and holes are clearly present. This is consistent with the action mechanism of azithromycin that inhibits protein synthesis by reversible binding to the 50S ribosomal subunits. Imamura et al. suggested in their study that deformation of the outer membrane could be produced by displacement of divalent cations, avoiding the binding of lipopolysaccharide (LPS) to its site of action [45].

In summary, it can be concluded that azithromycin microparticles maintain the antimicrobial activity of the pristine antibiotic, being effective against gram (+) as well as gram (−) bacteria.

### 3.5. Cytotoxicity Assay

The biocompatibility of the AZT formulation with human lung A549 and Calu-3 cells, from alveoli and bronchial epithelium, respectively, was evaluated by assessing the cellular metabolic activity (Alamar blue assay). Cell lines were exposed for 24 h and 48 h to AZT MPs or raw AZT at drug concentrations ranging from 0.1–1000 µg/mL.

**Figure 8:**
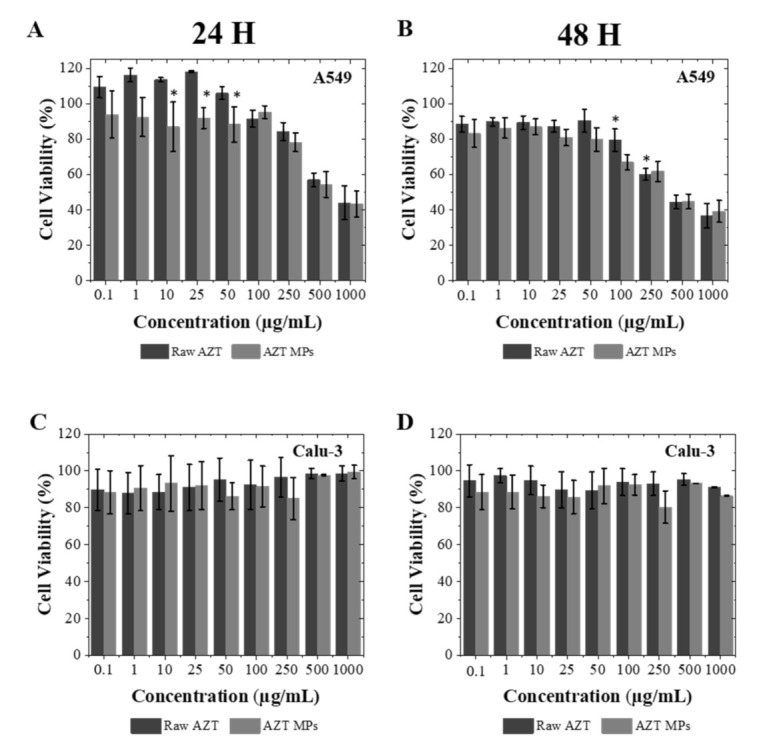
Cell viability measured by Alamar blue. A549 cell viability after (**A**) 24 h and (**B**) 48 h. Calu-3 cell viability after (**C**) 24 h and (**D**) 48 h. Both types of cells were exposed to increasing concentrations of microparticles in cell culture medium. Significant differences (*p* < 0.05) between different samples were tested in relation to raw AZT (mean ± SD, *n* = 3).

The results of the cytotoxicity assay showed that microparticles were well tolerated by Calu-3 (Figure 8C,D) and showed a slightly higher toxicity in A549, though only at elevated AZT MPs and raw AZT concentrations (Figure 8A,B). At 24 h, the viability of A549 decreased from microparticle concentrations of 250 µg/mL onwards and after 48 h still remained above 70% for concentrations up to 100 µg/mL, while the viability of Calu-3 was not affected. AZT MPs can then be considered cytocompatible for lung administration up to a concentration of 100 µg/mL in both cell lines, according to the international standard ISO 10993-5.

## 4. Conclusions

Electrospraying can be used to produce pure azithromycin microparticles in a suitable size for their use in DPI formulations. This technique yields excipient-free AZT microparticles in a simple, one-step procedure. The main objective of DPI formulations is to reach the lungs directly, thus being able to decrease the drug dose compared with other forms of administration. Having excipient-free particles is beneficial because it avoids the problems caused by excipients in certain groups of patients. The pristine size of the generated microparticles is around 2.5 µm, a value that would be suitable to reach deposition in lungs with a high yield value (85%). The deposition of these microparticles was evaluated in vitro for two different breathing profiles in COPD and healthy patients, and the methods predict that a similar dose would be inhaled in both cases. The antimicrobial activity against *S. aureus* and *P. aeruginosa*, two of the main bacteria causing infections in the human respiratory system, was also tested and similar MIC and MBC values were found, in comparison to those reported for azithromycin. Cytotoxicity assays performed on two different lines of human lung epithelial cells, A549 and Calu-3, demonstrated that AZT MPs are safe to be used at the MIC doses. In conclusion, electrospraying azithromycin appears as a suitable technique to produce excipient-free microparticles of a suitable size for direct pulmonary delivery using DPI devices. This formulation could expand the current treatment options for respiratory infections

## Figures and Tables

**Table 1:** Values obtained from the next generation impactor assays, (mean ± SD, *n* = 3).

	Constant Flow	Breath Profile	
	100 L/min	Healthy	Chronic Obstructive Pulmonary Disease (COPD)
Emitted Dose (ED) (%)	90.20 ± 0.40	86.65 ± 0.75	84.85 ± 0.80
Fine Particle Dose (FPD) (mg)	17.45 ± 1.00	12.55 ± 2.70	12.95 ± 3.35
Fine Particle Fraction (FPF) (%)	19.35 ± 1.05	14.50 ± 3.05	15.30 ± 4.05
Respirable Fraction (RF)	31.00 ± 4.15	29.10 ± 3.75	28.30 ± 4.10
Median Mass Aerodynamic Diameter (MMAD) (µm)	1.55 ± 0.08	1.56 ± 0.05	1.80 ± 0.10

**Table 2:** Particle distribution values of the aerosol cloud from AZT MPs by Spraytec^®^, (mean ± SD, *n* = 3).

Dv10 (µm)	Dv50 (µm)	Dv90 (µm)	Span
2.09 ± 0.06	9.96 ± 0.50	144.83 ± 16.95	14.330

## Data Availability

Not applicable.

## Supplementary Materials

The following are available online at https://www.mdpi.com/article/10.3390/pharmaceutics13121988/s1, Table S1: Determination of the inhaler Breezhaler^®^ resistance. The resistance was calculated by measurement of the flow rate for different values of the pressure drop (1, 2, 3 and 4 KPa) through the equipment. The resistance value was calculated as the slope of the linear fit of the square root of pressure drop versus flow rate *. Figure S1: Breathing profiles tested throughout the study. (a) Simulated respiratory profile of a healthy patient (total time: 2.51 s); (b) Simulated respiratory profile of a COPD patient (total time: 2.21 s). Table S2: Parameters and equations employed to obtain the breathing profiles. Figure S2: Experimental device for measurements with pre-determined breathing profiles. The set up consists of Alberta throat model^TM^, mixing inlet^TM^, breath simulator^TM^ and Next generation impactor (NGI)^TM^. Figure S3: EM images of particles generated for different solvents (AZT 10% *w*/*w*) the flow used was 1.5 mL/h. (a) ethanol; (b) ethanol/acetone (1/1); (c) acetone; (d) chloroform. Table S3: Solvent selection. Experimental parameters used (solvent, flow rate, AZT concentration) and resulting particle sizes. Table S4: Optimization of AZT concentration for electrospraying in chloroform. Experimental parameters used (concentration, flow rate) and resulting particle sizes. Figure S4: FTIR spectra of: (a) Raw azithromycin; (b) AZT MPs ethanol; (c) AZT MPs acetone; (d) AZT MPs chloroform. Figure S5: Differential Scanning Calorimetric: (a) raw Azithromycin; (b) electrosprayed microparticles. Figure S6: Characterization of aerosols formed by electrosprayed azithromycin microparticles. Data are shown by FPF and MMAD, *n* = 3, Mean ± SD, * *p* < 0.05. Figure S7: Particle size distribution of azithromycin particles as dry powder. Measurement was determined using a Spraytec at an airflow of 100 L/min (*n* = 3, data are represented as Mean ± SD). Figure S8: Antibacterial activity (CFU/mL) of AZT microparticles. (a) *S. aureus*; (b) *P. aeruginosa*, *n* = 3, Mean ± SD, * *p* < 0.05.
