# Supplementary material for: Excipient-Free Inhalable Microparticles of Azithromycin Produced by Electrospray: A Novel Approach to Direct Pulmonary Delivery of Antibiotics
Source: Pharmaceutics. 2021 Nov 23;13(12):1988. doi: 10.3390/pharmaceutics13121988 (PMC8704604; doi:10.3390/pharmaceutics13121988)
Supplement: Supplementary file 1 [file pharmaceutics-13-01988-s001.zip › pharmaceutics-1450162-supplementary.pdf]

pharmaceutics

Supplementary Materials: Excipient-Free Inhalable Microparticles of Azithromycin Produced by Electrospray: A Novel Approach to Direct Pulmonary Delivery of Antibiotics

Beatriz Arauzo, Tania B. Lopez-Mendez, Maria Pilar Lobera, Javier Calzada-Funes, Jose Luis Pedraz and Jesus Santamaria

Table S1. Determination of the inhaler Breezhaler® resistance. The resistance was calculated by measurement of the flow rate for different values of the pressure drop (1, 2, 3 and 4 KPa) through the equipment. The resistance value was calculated as the slope of the linear fit of the square root of pressure drop versus flow rate*.

Pressure Drop (KPa)	√Pressure Drop	Flow (L/min)
4	2	104
3	1.73	91
2	1.41	74.5
1	1	54.5
Device Resistance	0.0202 $KPa^{0.5}/L^{-1}min$	

* $y = 0.0202x - 0.101$ ($R^2 = 0.9997$).

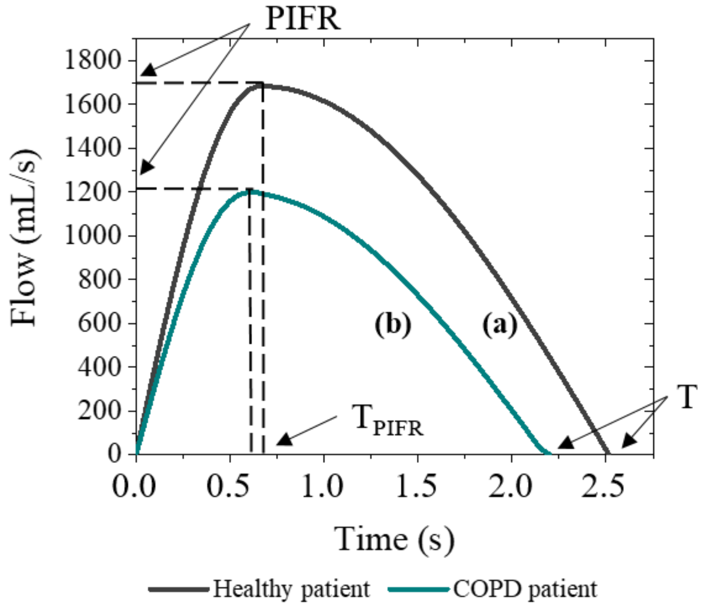

Figure S1. Breathing profiles tested throughout the study. (**a**) Simulated respiratory profile of a healthy patient (total time: 2.51 s); (**b**) Simulated respiratory profile of a COPD patient (total time: 2.21 s).

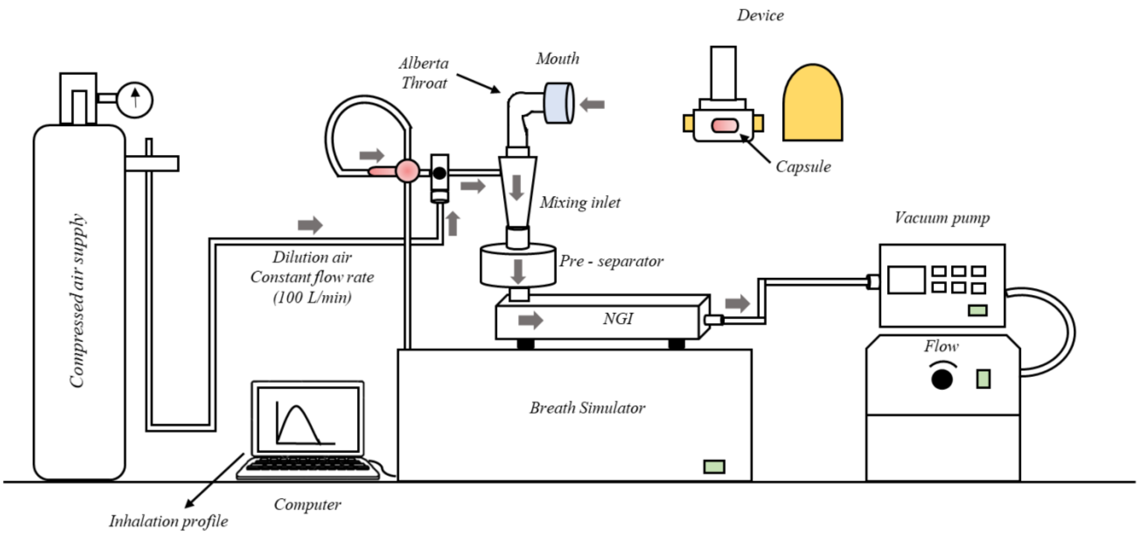

Figure S2. Experimental device for measurements with pre-determined breathing profiles. The set up consists of Alberta throat model™, mixing inlet™, breath simulator™ and Next generation impactor (NGI) ™.

Table S2. Parameters and equations employed to obtain the breathing profiles

Parameters*	Healthy Profile	COPD profile
PIFR	101.1 L/min	72 L/min
TPIFR	0.66 s	0.60 s
V	$T\ (s)\ = \frac{PIFR\ \times [L \times min^{-1}] \times T(s)}{30\ \pi}$	
	2.7 L	1.7 L
T_{total}	$T\ (s)\ = \frac{30\ \pi\ \times V(L)}{PIFR\ \times (L \times min - 1)}$	
	2.517 s	2.225 s
Function	$0 \le t < TIFR$<br>$FR\ (t) = PIFR\ \times \sin\ (\frac{\pi t}{2TPIFR})$<br>$TPIFR \le t \le T$<br>$FR\ (t) = PIFR\ \times \cos(\frac{\pi(t - TPIFR)}{2(T - TPIFR)})$	

*PIFR: Peak inspiratory flow rate; TPIFR: the time at which PIFR occurs; V: inhaled volume; T: Inspiration total time.

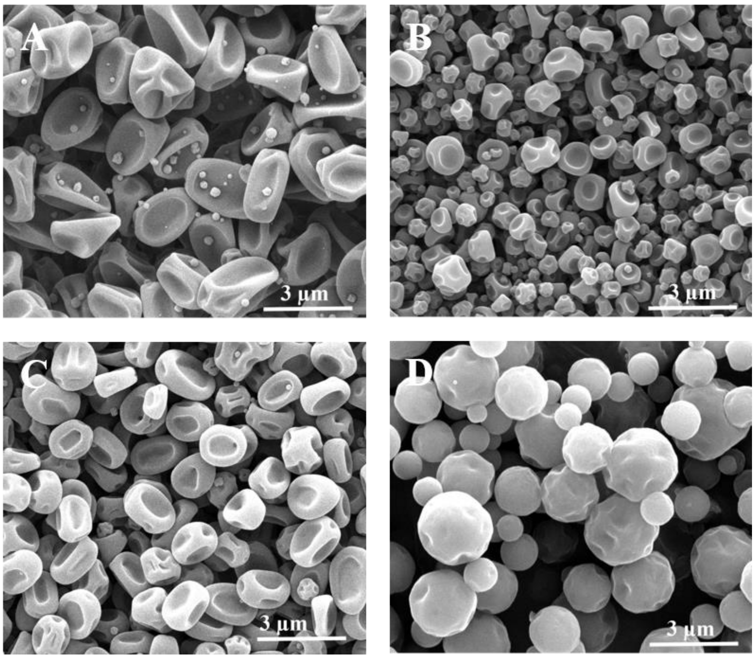

Figure S3. SEM images of particles generated for different solvents (AZT 10% *w*/*w*) the flow used was 1.5 mL/h. (**A**) ethanol; (**B**) ethanol/acetone (1/1); (**C**) acetone; (**D**) chloroform.

Table S3. Solvent selection. Experimental parameters used (solvent, flow rate, AZT concentration) and resulting particle sizes.

	**10% *w*/*w* Azithromycin**	
**Solvent**	**Flow (mL/h)**	**Size (µm)**
Chloroform	1	2.06 ± 0.75
	1.5	3.10 ± 0.60
	2	3.55 ± 0.90
	2.5	4.75 ± 0.75
Ethanol	1	1.40 ± 0.37
	1.5	2.30 ± 0.40
	2	1.80 ± 0.33
	2.5	2.00 ± 0.35
Acetone	1	1.10 ± 0.20
	1.5	1.15 ± 0.30
	2	1.65 ± 0.25
	2.5	0.90 ± 0.28
Ethanol/Acetone (1/1)	1	0.86 ± 0.26
	1.5	0.87 ± 0.27
	2	0.85 ± 0.22
	2.5	0.75 ± 0.30

Table S4. Optimization of AZT concentration for electrospraying in chloroform. Experimental parameters used (concentration, flow rate) and resulting particle sizes.

Chloroform		
**Concentration (% *w*/*w*)**	**Flow (mL/h)**	**Size (μm)**
1	1	1.07 ± 0.50
	1.5	1.10 ± 0.50
	2	2.50 ± 0.52
2.5	1	1.80 ± 0.72
	1.5	2.10 ± 0.70
	2	2.75 ± 1.03
5	1	1.70 ± 0.40
	1.5	2.90 ± 0.85
	2	2.40 ± 0.60
10	1	2.06 ± 0.75
	1.5	3.10 ± 0.60
	2	3.55 ± 0.90

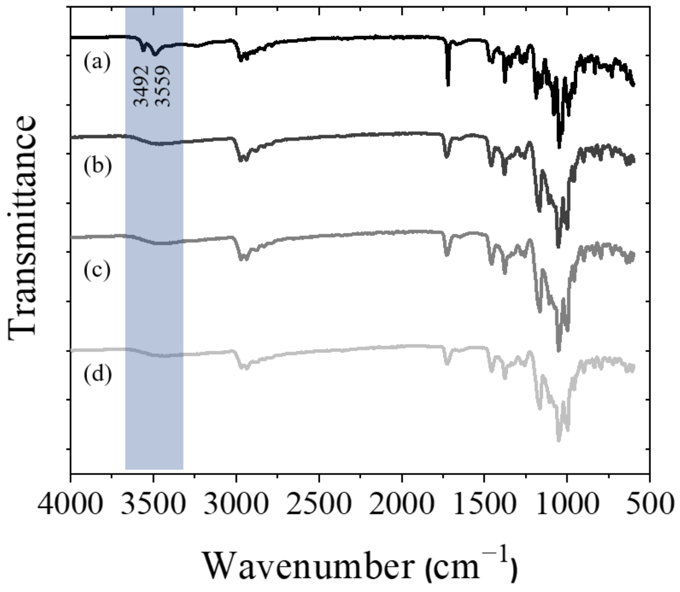

Figure S4. FTIR spectra of: (**a**) Raw azithromycin; (**b**) AZT MPs ethanol; (**c**) AZT MPs acetone; (**d**) AZT MPs chloroform.

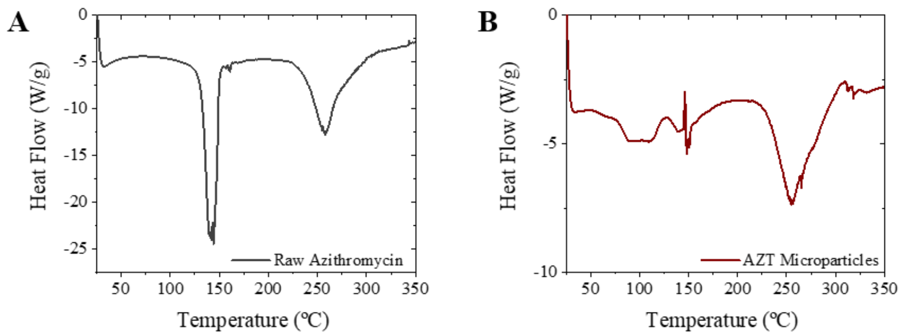

Figure S5. Differential Scanning Calorimetric: (**A**) raw Azithromycin; (**B**) electrosprayed microparticles.

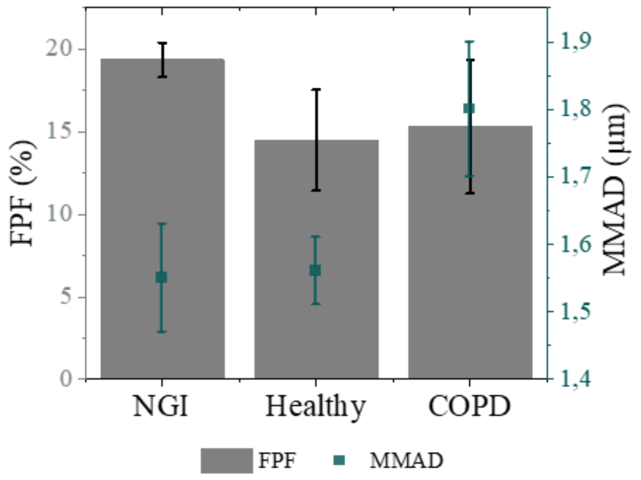

Figure S6. Characterization of aerosols formed by electrosprayed azithromycin microparticles. Data are shown by FPF and MMAD, n =3, Mean ± SD.

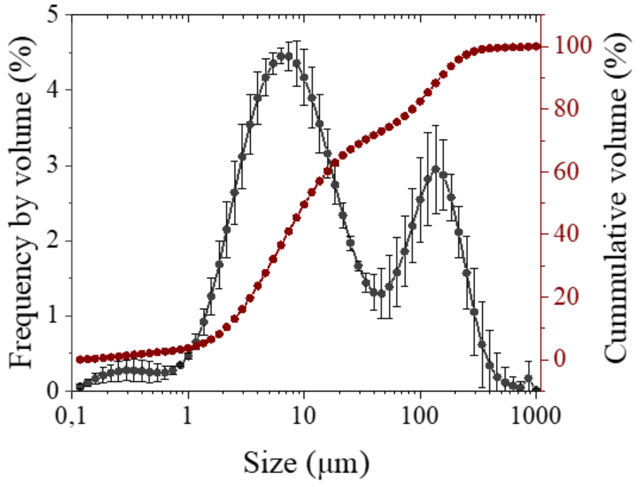

Figure S7. Particle size distribution of azithromycin particles as dry powder. Measurement was determined using a Spraytec at an airflow of 100 L/min (n = 3, data are represented as mean ± S.D.).

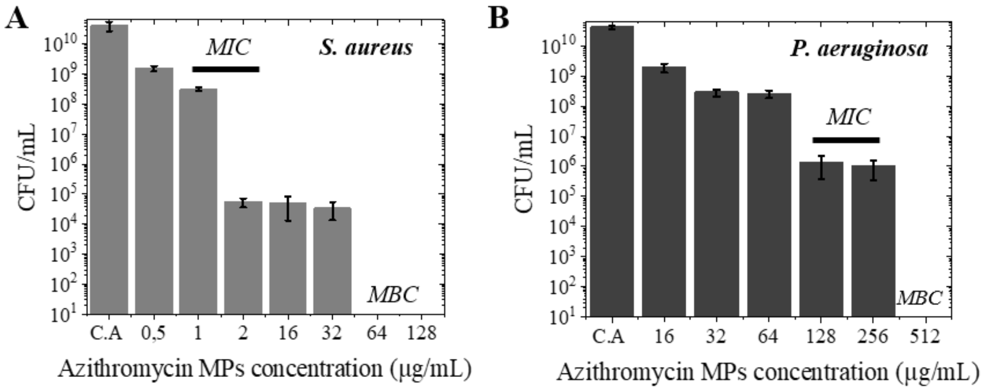

Figure S8. Antibacterial activity (CFU/mL) of AZT microparticles. (**A**) S. aureus; (**B**) P. aeruginosa, n =3, Mean ± SD.
